# ADAM10 and ADAM17: New Players in Trastuzumab Resistance

**DOI:** 10.18632/oncotarget.2794

**Published:** 2014-11-11

**Authors:** Michael J. Duffy, John Crown, Maeve Mullooly

**Affiliations:** Clinical Research Centre, St. Vincent's University Hospital, Dublin and UCD School of Medicine and Medical Science, Conway Institute, University College Dublin, Dublin, Ireland

The availability of the anti-HER2 monoclonal antibody, trastuzumab (Herceptin) has transformed the outcome of a subgroup of patients with breast cancer that previously had a poor prognosis, i.e., those with HER2-positive disease [1]. Although HER2 gene amplification/overexpression is necessary for breast cancer patients to receive trastuzumab, more than 50% of patients positive for this biomarker are intrinsically resistant to the treatment. Furthermore, almost all patients who initially respond, develop resistance which frequently occurs within the first year of treatment. Using mostly preclinical models, several different mechanisms have been proposed for conferring resistance to trastuzumab. These include hyperactivation of the PI3K pathway (due to PI3K mutations or PTEN loss), activation of alternative pathways (SRC, IGFR1), increased levels of EGFR/HER ligands and presence of HER2 isoforms [1].

In this issue of Oncotarget, Feldinger et al [2] describe a potential new mechanism of acquired resistance to trastuzumab, i.e., increased expression of ADAM10. ADAM10, together with the related ADAM17, are responsible for the release of all the ligands that bind to, and activate the EGFR/HER family of proteins [3]. Thus, ADAM17 is the primary sheddase for TGF-alpha, amphiregulin, HB-EGF, and epiregulin, while ADAM10 is believed to be primarily responsible for the release of EGF and betacellulin [3]. Previous evidence implicated high levels of these ligands in conferring resistance to anti-HER2 therapies, including trastuzumab [4,5].

Feldinger et al [2] now reports that trastuzumab increases ADAM10 levels in cell culture, in an animal xenograft model and importantly, also in patients. Furthermore, knockdown of ADAM10 or treatment with a selective low molecular weight ADAM10 inhibitor (INCB8765; Incyte) increased trastuzumab response in both naïve and trastuzumab-resistant HER2-positive cell lines. This enhanced response appeared to result from the inhibition of betacellulin release and subsequent reduced activation of EGFR, although this was not investigated in detail.

Consistent with these preclinical findings, the authors showed, using a small number of patients, that pretreatment ADAM10 levels were associated with a poor response and shorter relapse-free interval following treatment with trastuzumab. Previous research by the same group implicated increased levels of ADAM17 in conferring resistance to trastuzumab [6].

Taken together, these 2 reports [2,6] suggest that inhibition of ADAM10, ADAM17 or preferably both ADAMs is a potential new approach for minimizing resistance to trastuzumab. Currently, several ADAM10/17 inhibitors are available and indeed some of these have shown anti-cancer activity in preclinical systems [3,7]. To our knowledge, only one of these has undergone investigations in a clinical trial, for potential anti-cancer activity, i.e., the dual ADAM10/17 inhibitor, INCB7839 (Incyte) [8]. Preliminary results suggest that this drug is generally well tolerated with no major musculoskeletal side effects or anti-EGFR-related side effects such as skin rash. Furthermore, there were no reports of drug-induced increases in liver enzymes, bone marrow toxicity or increase in cardiomyopathy [8]. Evidence of target inhibition was the finding that administration of INCB7839 decreased shedding of different HER ligands as well as the extracellular domain of HER2.

The time has now come to further investigate ADAM10/17 inhibitors in animal models for minimizing resistance to trastuzumab. Hopefully, the preliminary results of Kong and colleagues [2,6] can be confirmed and that we can then move on to clinical trials using ADAM10/17 inhibitors in combination with trastuzumab.
